# Development of novel clinical examination scales for the measurement of disease severity in Creutzfeldt-Jakob disease

**DOI:** 10.1136/jnnp-2021-327722

**Published:** 2022-01-12

**Authors:** Akin Nihat, Tze How Mok, Hans Odd, Andrew Geoffrey Bourne Thompson, Diana Caine, Kirsty McNiven, Veronica O'Donnell, Selam Tesfamichael, Peter Rudge, John Collinge, Simon Mead

**Affiliations:** 1 UCL Institute of Prion Diseases, MRC Prion Unit at UCL, London, UK; 2 National Hospital for Neurology and Neurosurgery, University College London Hospitals NHS Foundation Trust, National Prion Clinic, London, UK

**Keywords:** scales, prion, dementia, motor control, cognition

## Abstract

**Objective:**

To use a robust statistical methodology to develop and validate clinical rating scales quantifying longitudinal motor and cognitive dysfunction in sporadic Creutzfeldt-Jakob disease (sCJD) at the bedside.

**Methods:**

Rasch analysis was used to iteratively construct interval scales measuring composite cognitive and motor dysfunction from pooled bedside neurocognitive examinations collected as part of the prospective National Prion Monitoring Cohort study, October 2008–December 2016.

A longitudinal clinical examination dataset constructed from 528 patients with sCJD, comprising 1030 Motor Scale and 757 Cognitive Scale scores over 130 patient-years of study, was used to demonstrate scale utility.

**Results:**

The Rasch-derived Motor Scale consists of 8 items, including assessments reliant on pyramidal, extrapyramidal and cerebellar systems. The Cognitive Scale comprises 6 items, and includes measures of executive function, language, visual perception and memory. Both scales are unidimensional, perform independently of age or gender and have excellent inter-rater reliability. They can be completed in minutes at the bedside, as part of a normal neurocognitive examination. A composite Examination Scale can be derived by averaging both scores. Several scale uses, in measuring longitudinal change, prognosis and phenotypic heterogeneity are illustrated.

**Conclusions:**

These two novel sCJD Motor and Cognitive Scales and the composite Examination Scale should prove useful to objectively measure phenotypic and clinical change in future clinical trials and for patient stratification. This statistical approach can help to overcome obstacles to assessing clinical change in rapidly progressive, multisystem conditions with limited longitudinal follow-up.

## Introduction

The human prion diseases are a group of universally fatal neurodegenerative conditions caused by the autocatalytic, templated misfolding of the constitutively expressed prion protein (PrP^C^) into disease-related assemblies, including protease-resistant forms designated PrP^Sc^.^1^ They can be separated aetiologically into sporadic (approximately 85% of incident cases), inherited (10%–15%) and acquired (<1%) subtypes. The median clinical duration from symptom onset in sporadic Creutzfeldt-Jakob disease (sCJD) is 4 months, although disease courses ranging from short weeks to several years are recognised.^2 3^

Even within aetiological groups there is enormous heterogeneity in clinical phenotype,^3–5^ duration and histopathology, partly reflecting the propagation of distinct conformational strains of misfolded prion protein.^1^ Well-designed clinical rating scales are necessary to interpret and manage this variability, by providing quantitative measures of disease-specific change, as clinical trial eligibility and stratification criteria, outcome measures or for prognostic modelling.^6^ They may also provide useful information for patients, caregivers and healthcare professionals about aspects of disease progression, specific functions or symptoms.

In 2013, we proposed the Medical Research Council Prion Disease Rating Scale (MRC Scale),^7^ a validated functional outcome measure of disease progression in sCJD, acquired by brief carer interview. The MRC Scale score encapsulates a patient’s functional performance, and allows direct group and individual comparison of disease progression in a clinically and statistically meaningful manner,^6^ and usefully, can be acquired remotely.

The MRC Scale was developed using Rasch modelling, a form of item-response modelling that offers considerable advantages over scales constructed using ‘classical test theory’^6 8^—particularly in populations with a high degree of missing or complex longitudinal data, such as rapidly progressive dementias, or to combine multiple complementary scales. Crucially, Rasch-derived scales are unidimensional and linear^9^: all components measure the same trait and form a true interval scale—for example, a difference in one point represents the same degree of change regardless of the total score; these properties are not true of classical test theory-developed scales,^10^ such as the Mini-Mental State Examination (MMSE).^11^

Despite its utility and widespread use, the MRC Scale does not directly measure the impairments that contribute to functional deficits, and relies on a witness report. No clinical rating scales have been developed that specifically and robustly measure progressive cognitive or motor dysfunction in sCJD—previous attempts have been constrained by the wide phenotypic diversity and rapid clinical change,^12^ and use of clinical scales developed for generic cognitive or motor impairment is suboptimal in a clinical trials setting.^13^

We therefore sought to use Rasch modelling to develop physician examination-orientated neurological and cognitive rating scales, complementing the functionally oriented MRC Scale as clinimetric tools, and demonstrating a model to develop similar tools in other complex dementia syndromes.

## Methods

### Study population

The National Prion Monitoring Cohort (NPMC) protocol is published^7^; at enrolment, each patient has a comprehensive clinical assessment, including a standardised neurological examination and bedside neuropsychological battery (Short Cognitive Exam (SCE)^14^), MMSE and the MRC Scale.^7^ Assessments are repeated at domiciliary visits at 4–8 weeks intervals, depending on rate of clinical change, and the MRC Scale is repeated remotely every 2 weeks.

The scale development population initially included all patients with a clinical diagnosis of probable sCJD^15^ recruited to the NPMC between October 2008 and December 2016 (total n=430, pathologically confirmed diagnosis in 231; 54%). For the Motor Scale, this was reduced to those patients recruited between July 2013 and December 2016 (n=168, 78 pathologically confirmed; 46%)—reflecting the time at which key assessments of cerebellar function were added to the NPMC: the Scale for the Assessment and Rating of Ataxia (SARA)^16^ and the Composite Cerebellar Functional Severity score,^17^ components of which became incorporated in the Motor Scale developed here.

Cognitive and Motor Scale scores and cognitive/motor ratios were subsequently calculated for all other patients with any form of prion disease recruited to the NPMC between October 2008 and December 2016.

### Item bank development

Two ‘item banks’ were created from routine elements of the standardised examination, or other pre-existing scales that could reflect motor or cognitive function. Each examination component, or ‘item’ (eg, upper limb ataxia, forward digit span), was documented as a numeric, ordinal score.

The motor item bank was designed to include items dependent on the integrity of the pyramidal and extrapyramidal motor systems and voluntary motor coordination, frequently affected in sCJD.^15^ Additional composite or functional items that could also capture impairment of one or more motor pathways were included, such as eye movements, gait and mobility.

The cognitive item bank was initially composed of items from the Short Cognitive Exam. In addition, to increase face validity, we gave particular weight to items that assessed domains particularly affected in prion disease, which comprises a global dementia with predominant frontoparietal impairment.^14^ Major challenges of monitoring cognitive function in rapidly progressive dementias such as sCJD include the attrition rate of patients who are only able to complete one or two longitudinal assessments prior to entering an advanced disease stage,^13^ and patient fatigue—necessitating a brief assessment tool able to measure differences between severely impaired or dysphasic patients. We sought to mitigate this by limiting the final scale to six items, and including in the final bank those items with the greatest pooled standardised effect size between two sequential patient assessments, to maximise the ability to stratify patients over the minimum number of assessments.

### Scale development using Rasch analysis

We used the partial credit form of polytomous Rasch analysis (RUMM2030, standard edition) to iteratively refine each item bank into a unidimensional, interval scale reflecting different degrees of composite motor or cognitive dysfunction in sCJD.

Fit to the Rasch model was assessed using a number of approaches,^18^ including: threshold ordering (each item’s scores progress in the expected order); item-trait and item-person interactions (the items are ordered by difficulty, and score does not depend on measurement conditions); local dependencies (the score in one item does not depend on the score in another); differential item functioning (scale scores are not affected by age, gender or codon 129). Scale inter-rater reliability was assessed using intraclass correlation (ICC) coefficients (Van de Winckel *et al* 19) for independent prospective Motor and Cognitive Scale scores for 30 consecutive patients, undertaken by either a doctor or specialist nurse.

### Data imputation and further statistical analysis

Rasch-derived scales encapsulate information on both the subject’s ability and the difficulty of all item thresholds, so a partial score can be used to impute the remaining components.^20^

We therefore used the Rasch-derived Motor and Cognitive Scales to impute total scale scores where some of the scale items were missing at random. For the Motor Scale, this comprised all symptomatic patients recruited to the NPMC between October 2008 and June 2013, prior to the introduction of the SARA to the standardised assessment protocol.

We used the Cognitive Scale to impute missing data where no more than 2/6 scale items or 10/20 of total possible score were missing. Patients with an MRC Scale score below 5/20 are generally bedbound, have little awareness of surroundings, are unable to use tools and communicate in single words at best.^7^ We therefore imputed a missing Cognitive Scale score of 0 for patients with an MRC Scale score below 5 and MMSE score of 0. To validate this approach, we collected prospective Cognitive and Motor Scale scores in newly enrolled patients with prion disease. In 30 consecutive patients with sCJD with MRC Scale scores below 5/20 (mean 3.0/20, SD ±0.84), the mean Cognitive Scale score was 0.3/20 (SD ±0.91).

## Results

### Patient selection for scale development

One hundred and six patients recruited between July 2013 and December 2016 were included in Rasch analysis to develop the Motor Scale (table 1), comprising 144 individual assessments. Sixty-two additional patients were excluded from analysis due to missing examination data; these patients were at advanced neurodisability (maximum group MRC Scale score 4/20), precluding a formal neurological examination beyond assessment of consciousness or limb tone. We reasoned that outcome measures aimed at stratifying motor function in patients at such advanced disability would not be clinically meaningful, nor possible given a dearth of clinical data. All included patients were diagnosed as having probable sCJD by consensus criteria.^15^ Forty-eight (45%) underwent postmortem, confirming the diagnosis in all cases.

**Table 1:** Demographic characteristics for patients included in Motor and Cognitive Scale development cohorts, all of whom had a clinical diagnosis of sCJD

	Motor Scale development cohort	Cognitive Scale development cohort
No. of patients (assessments)	106 (144)	155 (231)
Mean age (SD)	67.0 (7.4)	64.5 (8.6)
Gender M/F	52/54	93/62
Codon 129		
MM (%)	30 (28.0)	28 (18.1)
MV (%)	26 (24.5)	45 (29.0)
VV (%)	29 (27.3)	54 (34.8)
Unknown (%)	21 (19.8)	28 (18.1)
Mean MRC Scale score at assessment/20 (range, SD)	8.6 (20–0, 5.2)	11.6 (20–0, 4.2)

MM, MV, VV, codon 129 genotypes at PRNP; MRC, Medical Research Council; sCJD, sporadic Creutzfeldt-Jakob disease.

Two hundred and thirty-one assessments from 155 unique patients were included in the Cognitive Scale development (table 1). Seventy-seven (50%) patients underwent postmortem examination, confirming the diagnosis in all cases. Two hundred and seventy-five additional patients were excluded from the scale development cohort, the majority due to advanced disability, which precluded formal cognitive assessment using the SCE.

### Motor Scale development

An initial bank of 20 items was pooled as a single scale, but demonstrated poor fit to the Rasch model. Items were iteratively altered or removed according to threshold order, item and person trait interactions, co-dependency and differential item functioning.

**Figure 1:**
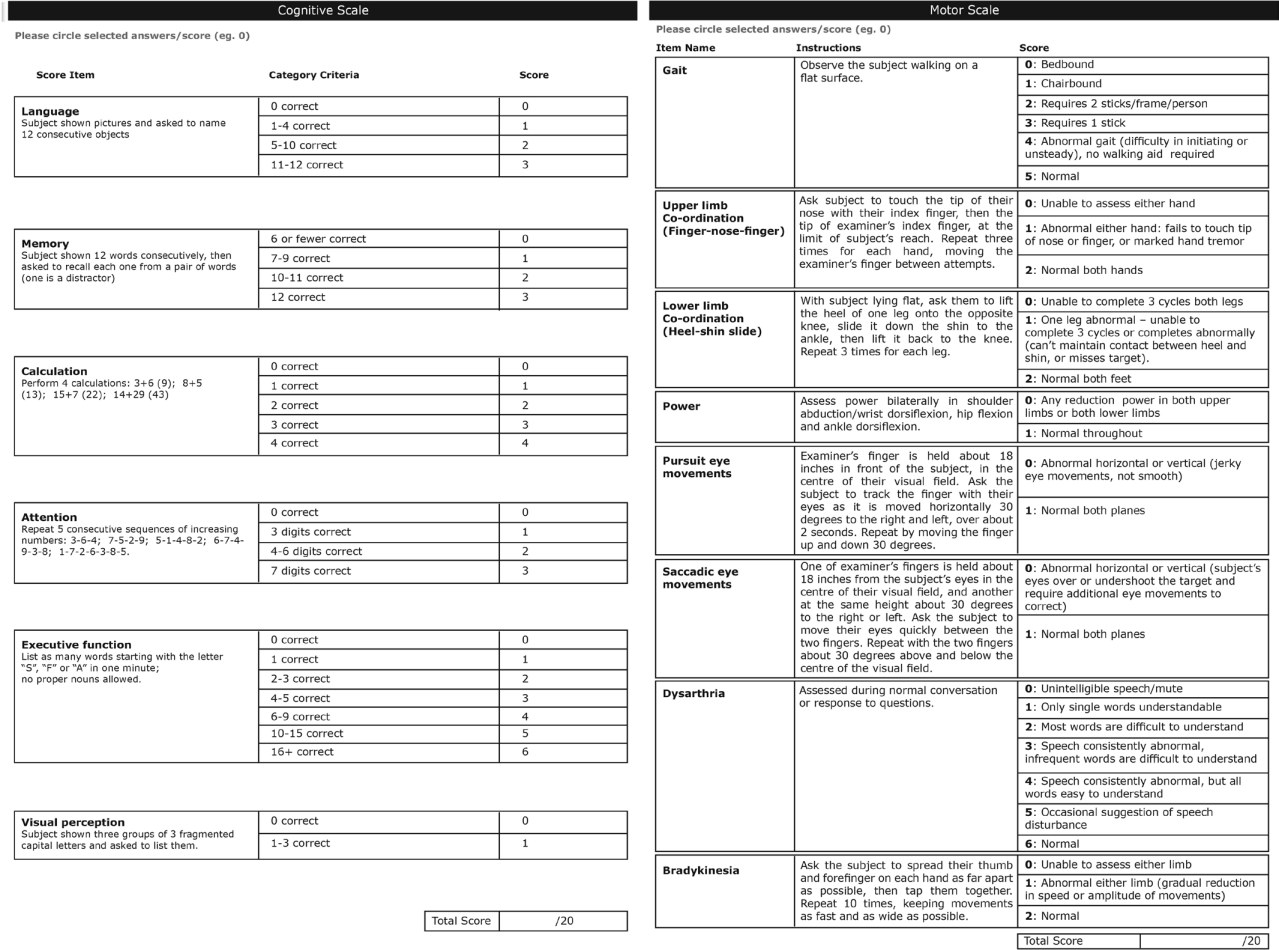
Final Rasch-derived Cognitive and Motor Scales, including instructions and scoring.

The final, 8-item, 20-point MRC Prion Disease Motor Scale (Motor Scale) (figure 1) demonstrated good overall fit to the Rasch model. The person separation index was 0.72 (where values >0.70 are acceptable^21^) and χ^2^=27.2 (df=24, p=0.29), without local dependence between items or differential item functioning (for age, codon 129 or gender). A mean item fit residual of −0.28 (SD ±1.00) and person fit residual of 0.32 (SD ±0.86) implied both scale items and patients fit the Rasch model. The final Motor Scale included items reliant on pyramidal, extrapyramidal and cerebellar systems, in addition to gait, which is reliant on multiple motor systems. Items removed due to poor ability to discriminate patients at different stages of motor progression included the presence and severity of myoclonus, assessment of limb tone and deep tendon reflexes. The ICC coefficient (one-way random effects model) was 0.98 (95% CI 0.96 to 0.99), indicating excellent inter-rater reliability.

**Figure 2:**
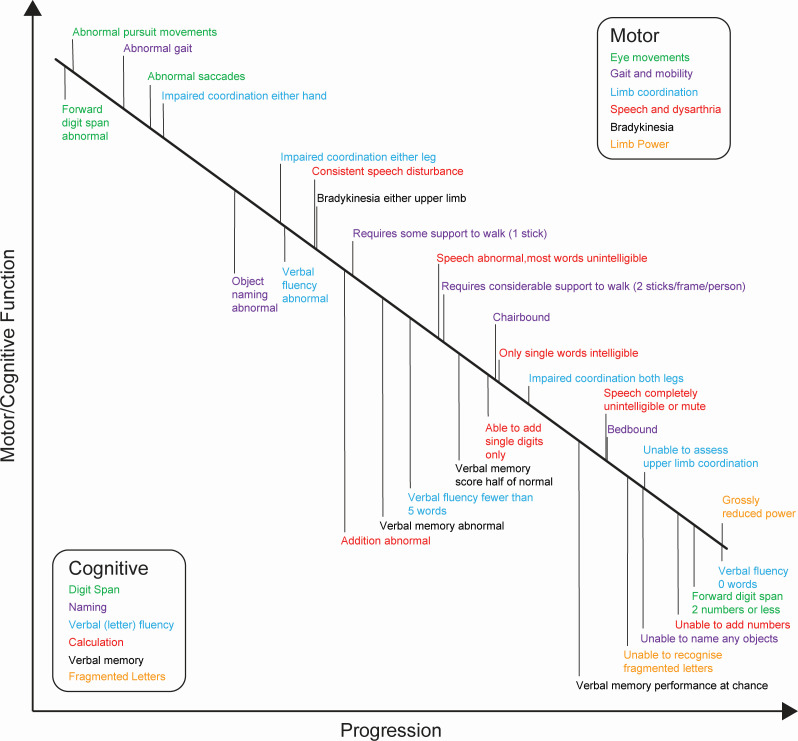
Typical pattern of progressive motor (above the line) and cognitive (below the line) dysfunction in prion disease, according to the Rasch model.

The Rasch model permits ordering of items and their component thresholds according to their hierarchical difficulty, reflecting composite ability in a desired trait. The final Motor Scale can therefore be used to infer the relative pattern of progression in motor dysfunction for patients with sCJD, as demonstrated in figure 2. Among the earliest motor features in the natural history of sCJD are impaired saccadic and smooth pursuit eye movements, followed by abnormal gait. Conversely, grossly reduced limb power is a relatively late feature.

### Cognitive Scale development

The initial cognitive item bank included assessment of executive function, language, parietal lobe function, visual perception, recognition memory, attention and praxis.^14^

Standardised effect sizes for each item were calculated for 56 patients with two serial assessments, ranging from 0.55 (calculation) to 0.24 (reading). Spelling and reading items were excluded from the item bank due to low standardised effect sizes, and overlap with other items in the cognitive domains they assessed (parietal lobe function and language, respectively). The remaining items were scaled where necessary into ordinal bins to ensure similar total scores for each item. An initial composite scale demonstrated reasonable fit to the Rasch model as judged by mean item and person fit residuals, but inadequate ability to discriminate patients (person separation index 0.66) and no item invariance (χ^2^=32.5, df=18, p=0.02). The item bank was iteratively refined; all three praxis tasks were removed, having demonstrated poor individual ability to discriminate patients and overall model fit improved. Visual recognition was removed due to local dependence with verbal recognition.

The final MRC Prion Disease Cognitive Scale (Cognitive Scale) comprised six items, including measures of executive function, language, parietal lobe function, visual perception, recognition memory and attention/working memory (figure 1). It displayed good fit to the Rasch model: an acceptable ability to discriminate patients (person separation index 0.74); item invariance (total item χ^2^=21.1, df=18, p=0.27); good item fit (mean fit residual 0.07, SD ±0.92) and person fit (mean fit residual −0.45, SD ±0.99). There were no local dependencies or differential item functioning (for age, codon 129 or gender). The ICC coefficient was 0.98 (95% CI 0.95 to 0.99).

The Rasch-derived schematic pattern of progressive cognitive dysfunction in sCJD implies earlier deficits in language (naming) and executive function (verbal fluency), with deficits in recognition memory and visual perception occurring later (figure 2).

### Using Cognitive and Motor Scales to quantitively demonstrate phenotypic heterogeneity

Cognitive and Motor Scale scores for the entire NPMC (including patients with sCJD, variant and iatrogenic forms and inherited prion disease) were calculated using three approaches: prospectively assessed scores using the novel scales, from October 2018 to August 2019 (Motor n=182, Cognitive n=177); scores calculated retrospectively from items included in the final Rasch-derived scales (Motor n=293, Cognitive n=361); imputing missing components of Cognitive and Motor Scale scores as outlined in ‘Methods’ section (Motor n=1403, Cognitive n=833). In total, 528 patients with sCJD had at least one Cognitive or Motor Scale score, and 435 patients with sCJD had at least one paired score (both Cognitive and Motor scores at the same clinical visit).

**Figure 3:**
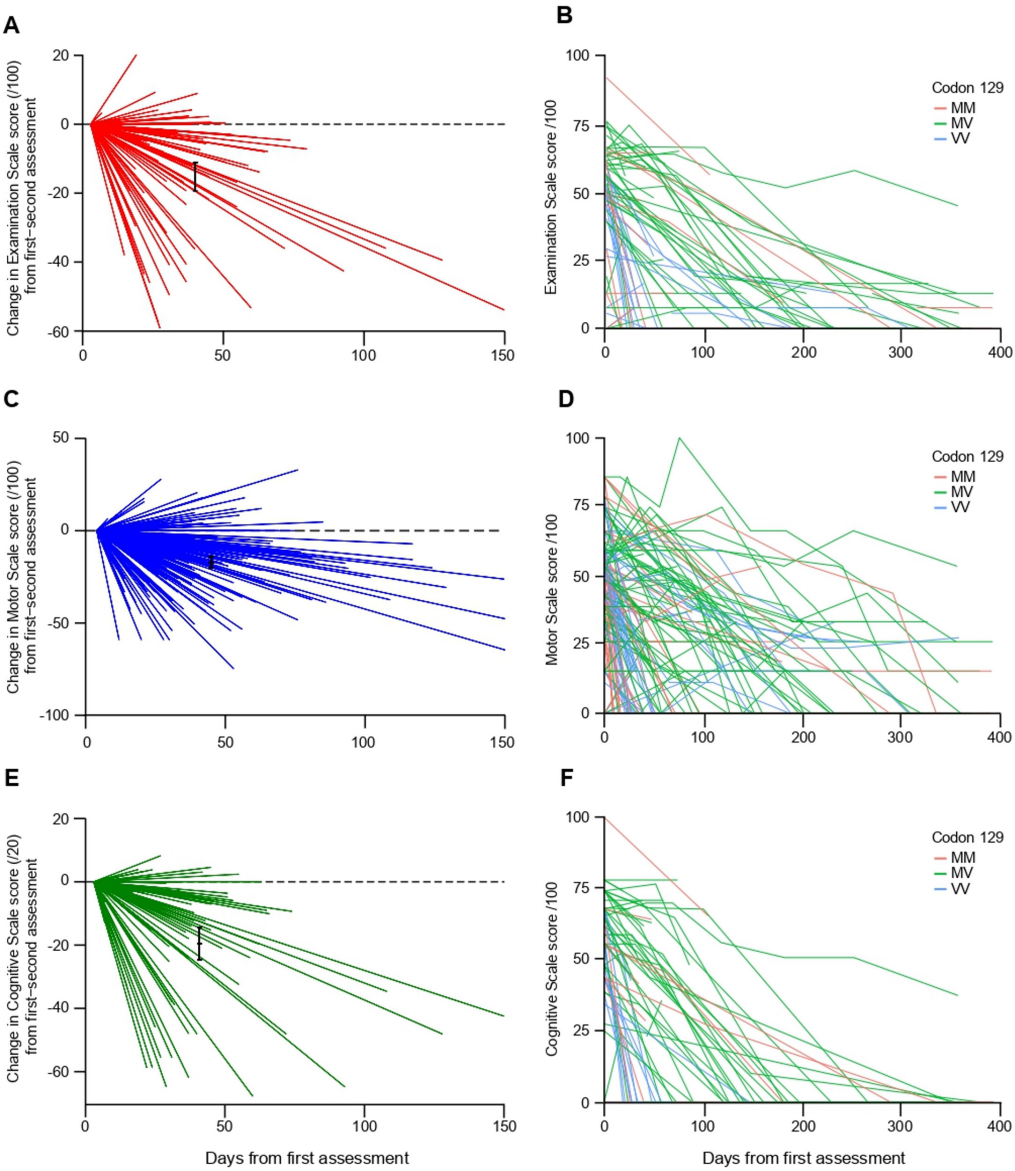
Longitudinal change in Motor and Cognitive Scales between first and second assessment. Change in Examination Scale (A), Motor Scale (C) and Cognitive Scale (E) score between first (anchored to score of 0) and second assessment for individual patients with sporadic Creutzfeldt-Jakob disease (sCJD), /100; reference line indicates no change between assessments, mean change (95% CI) and mean follow-up date in black. Spaghetti plots of individual sCJD patient trajectories for Examination (B) Motor (D) and Cognitive (F) Scale scores over multiple assessments, grouped by PRNP codon 129 polymorphism.

Individual Cognitive and Motor Scale longitudinal patient trajectories suggested an approximately linear decline in function over time in most patients (figure 3). Higher Cognitive or Motor Scale scores at enrolment were associated with longer subsequent survival in patients with sCJD (Spearman’s rho: Cognitive Scale=0.46, p<0.00001; Motor Scale=0.51, p<0.00001).

We estimated a simple least-squares linear regression model of each patient’s first Cognitive and Motor Scale score with paired MRC Scale scores, to evaluate the proportion of variance in Cognitive and Motor function explained by overall disease progression. Changes in the MRC Scale explained 72% of variance in Cognitive score (model *F* (1, 402)=1052.4, p<0.00001) and 80% in Motor score (model *F* (1, 496)=1961.0, p<0.00001). We estimated that a Cognitive Scale score of 1/20 was reached at MRC Scale score=3.9 (95% CI 3.6 to 4.3) and Motor Scale score 1/20 at MRC Scale score 3.5 (95% CI 3.2 to 3.7), indicating expected floor effects at this level, when most patients are bedbound, unable to verbalise and without awareness of their surroundings.^7^

**Figure 4:**
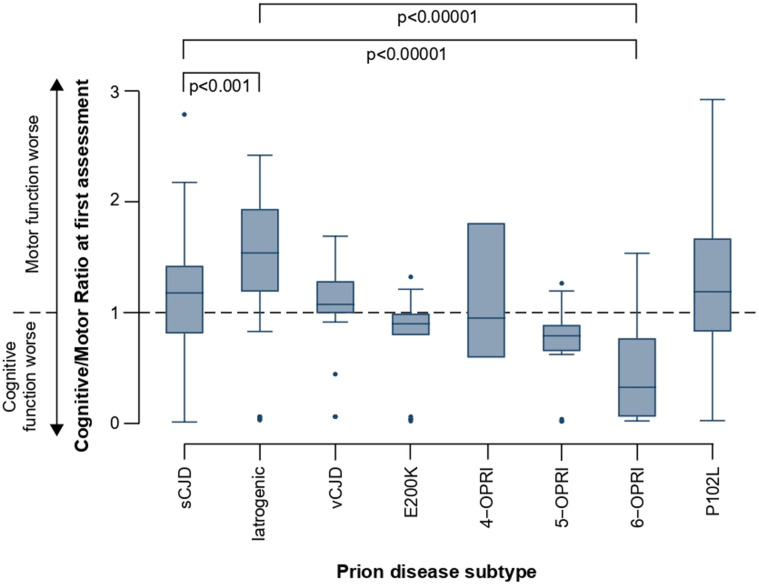
Cognitive/Motor scale score ratios (CM ratios) at first assessment for different prion disease aetiologies. Displayed are median scores, IQRs (boxes) and limits of Q1/Q3+1.5×IQR (whiskers). Subtypes were compared using the Kruskal-Wallis test for non-parametric data, followed by ad hoc pairwise comparison with Dunn’s test, adjusted for multiple comparisons. sCJD=sporadic Creutzfeldt-Jakob disease, n=435; iatrogenic=iatrogenic CJD, n=18; vCJD=variant CJD, n=12; inherited prion disease (E200K mutation), n=19; inherited prion disease (4-OPRI mutation), n=4; inherited prion disease (5-OPRI mutation), n=8; inherited prion disease (6-OPRI mutation), n=20; inherited prion disease (P102L mutation), n=29—patients with a CJD phenotype, n=5^40^ are marked as orange circles. Differences shown here illustrate the face validity of our scales.

**Figure 5:**
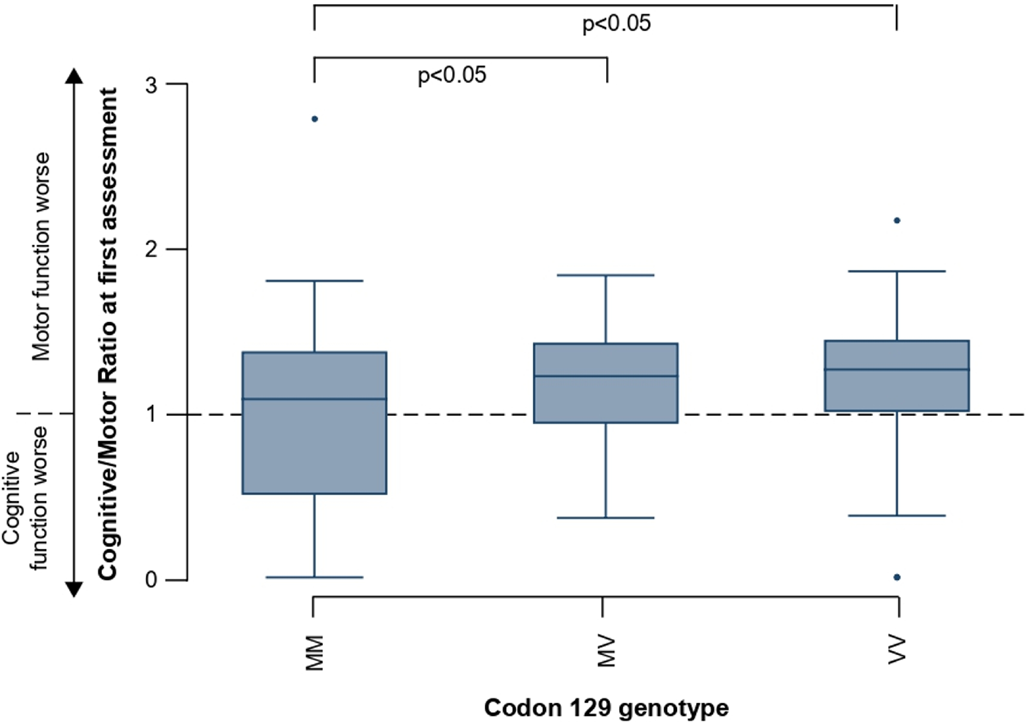
Cognitive/Motor Scale score ratios (CM ratios) at first assessment for patients with sporadic Creutzfeldt-Jakob disease (sCJD) according to codon 129 polymorphism. Displayed are median scores, IQR (boxes) and limits of Q1/Q3+1.5×IQR (whiskers). Subtypes were compared using the Kruskal-Wallis test for non-parametric data, followed by ad hoc pairwise comparison with Dunn’s test, adjusted for multiple comparisons.

Finally, we explored the potential for paired Cognitive and Motor Scale scores to quantitatively reflect phenotypes between prion disease subtypes, which result in different patterns of cognitive and motor dysfunction.^4^ We calculated Cognitive/Motor Scale score ratios (CM ratio) for patients with at least one pair of assessments (total n=570, sCJD n=435; table 2), where a CM ratio of >1 indicates greater relative motor dysfunction; and <1, greater relative cognitive dysfunction. We compared the first available CM ratio in patients with an MRC Scale score >4/20, for patients according to prion disease subtype (figure 4), and across codon 129 genotype in patients with sCJD (figure 5).

**Table 2:** Cognitive/Motor ratio cohort demographics

Variable	All patients (all prion aetiologies)					Patients with sCJD				
	No. of patients (total paired records)	No. with MRC (total paired records)	Mean first assessment MRC±SD (95% CI)	Mean first assessment Motor100*±SD (95% CI)	Mean first assessment Cog100*±SD (95% CI)	No. of patients (records)	No. with MRC (records)	Mean first assessment MRC±SD (95% CI)	Mean first assessment Motor100*±SD (95% CI)	Mean first assessment Cog100±SD (95% CI)
At least one paired Motor and Cognitive Scale score	570 (1286)	545 (1169)	7.4±6.9 (6.8 to 8.0)	30.3±29.4 (27.9 to 32.8)	27.0±31.8 (24.4 to 29.7)	435 (728)	427 (690)	6.1±6.1 (5.5 to 6.7)	24.6±26.0 (21.1 to 27.1)	22.0±29.5 (19.2 to 24.8)
Aetiology										
sCJD	435 (76.3)					435 (100.0)				
vCJD	12 (2.1)									
Inherited	104 (18.2)									
Iatrogenic	19 (3.3)									
Gender M/F	71/64					201/234				
Mean age (SD)	45.7 (12.4)					66.4 (9.1)				
Codon 129										
MM (%)	252 (44.2)					178 (40.9)				
MV (%)	150 (26.3)					103 (23.7)				
VV (%)	97 (17.0)					89 (20.5)				
Unknown (%)	71 (12.5)					65 (14.9)				

*Motor and Cognitive Scale scores transformed to scores/100 according to the Rasch model.

MRC, Medical Research Council; sCJD, sporadic Creutzfeldt-Jakob disease; vCJD, variant CJD.

Patients with iatrogenic CJD (iCJD) due to treatment with contaminated cadaveric growth hormone typically present with early cerebellar ataxia and dysarthria, but relatively preserved cognition.^22^ This is reflected in the median CM ratio of patients with iCJD, which is significantly greater than those with sCJD (Kruskal-Wallis/Dunn’s post hoc pairwise comparison p<0.00001). Conversely, patients with inherited prion disease caused by the six octapeptide repeat mutation (6-OPRI) manifest a prominent cortical dementia, with onset of motor system dysfunction occurring much later^23^—as evidenced by a low CM ratio. The CM ratio may also be valuable in objectively quantifying the phenotypic progression in inherited prion diseases, most of which have a disease course spanning years and can manifest dominant motor or cognitive phenotypes. For example, the predominantly cognitive phenotype^24^ of the 6-OPRI mutation is reflected in a very low CM ratio, while the P102L mutation group usually have a leading cerebellar ataxia, as evidenced by its median CM ratio >1. Interestingly, the P201L mutation can present with rarer cognitive dominant or sCJD-like phenotypes,^25^ and these differences are reflected by the mean CM ratio of patients across the postenrolment disease course (online supplemental figure 1).

10.1136/jnnp-2021-327722.supp1 Supplementary data

Among patients with sCJD, the median CM ratio differed according to codon 129 genotype. The median CM ratio for patients homozygous for methionine at codon 129 was significantly lower than those heterozygous or valine homozygous, indicating greater relative motor dysfunction in the latter two groups (figure 5). Twenty-six patients were classified by postmortem molecular PrP^Sc^ typing under the London classification,^3^ which is broadly comparable to another commonly used classification system.^4 26^ Patients with *PRNP* 129 VV genotype and PrP^Sc^ type 3 (Parchi VV2) present with striking ataxia,^4^ and within this study their mean first CM ratio was 1.41 (SD ±0.36), indicating greater relative motor dysfunction; those with *PRNP* 129 MM genotype and PrP^Sc^ type 2 (London classification; Parchi MM1) experience dominant early cognitive dysfunction,^4^ reflected by their mean CM ratio of 0.94 (SD ±0.90), indicating predominant cognitive dysfunction at first assessment.

### The composite Examination Scale score

The Cognitive and Motor Scales provide information about different aspects of a patient’s clinical progression at the bedside. We sought to also encapsulate this change in a single score that could provide a measure of overall progression on bedside clinical examination—the Examination Scale. This was derived simply as an average of each paired Cognitive and Motor Scale score taken at the same time, for 528 patients with sCJD with at least one Cognitive or Motor Scale score.

Examination Scale score sCJD patient trajectories (figure 3C-F) generally showed a longitudinal linear decline, and higher scores at presentation were associated with longer subsequent survival (Spearman’s rho=0.34, p<0.00001).

## Discussion

We have used Rasch modelling to develop two validated, disease-specific, clinician examination-based rating scales for patients with sCJD, reflecting disease progression in cognitive and motor systems. Both scales can be completed in minutes, avoid jargon or abstract scoring systems and require no special equipment. They use items commonly assessed during a routine neurological and bedside cognitive examination. A composite Examination Scale score provides a single measure of clinical progression on bedside examination. These scales complement the functionally orientated MRC Scale and have several potential uses.

Objectively quantifying motor and cognitive dysfunction in prion disease should add valuable clinimetric tools for patient assessment both in formal clinical trials and routine neurological practice. Differences in the speed and extent of motor versus cognitive dysfunction can help to stratify patient phenotypes that progress at different rates,^27^ and thus provide more accurate prognostic information for patients and their families. Moreover, documenting a patient’s motor or cognitive function using these scales will allow any clinician to make inferences regarding the likely future sequence and pace of clinical change, and thus allow more effective planning of future care needs and information provision.^28^ Informal pilot use of both scales via telemedicine has proven feasible with a local clinician able to examine the patient, and could allow remote assessment in future practice.

Previous efforts to develop system-specific clinical rating scales for prion disease therapeutic trials have attempted to grade individual neurological examination features, such as degree of ataxia, or total accretion of neurological signs.^12 29^ These approaches suffer with the issues that warranted our use of Rasch methodology: they do not measure a single global or composite construct, are not validated interval scores and are dependent on the population in which they are measured; thus comparing scores between or within patients or groups is unreliable.^6^ Similarly, cognitive assessment in prion disease has traditionally used the MMSE, which does not assess executive function^30^—commonly impaired in prion disease^14 31^—and the Clinical Dementia Rating scale, which is time-intensive to administer, requires training and is heavily dependent on carer interview.^32^ We have previously shown that these broad cognitive tests are ineffective at stratifying cognitive function in patients with sCJD.^13^

The key elements of each final scale were broadly consistent with clinical signs and symptoms most prominent in prion disease. The Motor Scale predominantly involves assessment of cerebellar function, which is impaired at presentation in most patients with sCJD.^4^ Conversely, extrapyramidal dysfunction constitutes a small portion of the Motor Scale and is generally observed later in the disease course.^33^ We found that altered limb tone and deep tendon reflexes are not useful measures to stratify motor dysfunction in prion disease. Interestingly, despite its textbook association with prion disease and observation as part of most clinical phenotypes,^33^ myoclonus was also not useful in measuring motor dysfunction. This is perhaps unsurprising given its binary classification, occasional spontaneous remission with disease progression and abundance of effective treatment options.^34^

The Cognitive Scale supports leading executive dysfunction and expressive language impairment in prion disease, with recognition memory a less prominent component.^14^ The Heidenhain variant phenotype is a well-documented presentation of sCJD involving higher order visual dysfunction at onset; visual perception was affected late in the Cognitive Scale, and was a small contributor to progression. This is likely to reflect the strength and limitation of Rasch-derived scales: by definition, scales are a composite of the most useful items to reflect the entire span of the desired trait—thus, items that assess rare symptoms or signs have little value in stratifying most patients, and are likely to be excluded. Although one of the most commonly discussed clinical phenotypes, the Heidenhain variant affects only about 5% of patients with sCJD.^35^

These scales also allow quantification of relative cognitive versus motor dysfunction in individual patients for the first time, and potentially a means to objectively measure and track different clinical phenotypes. An initial investigation in a small group of patients suggests the CM ratio does reflect described motor or cognitive-dominant phenotypes and molecular PrP^Sc^ types, Of note, we compared the first available CM ratio between patients, which may change during the disease course; however, comparison of the CM ratio between P102L mutation patients across the disease course appears to support their use to objectively separate clinical phenotypes. A larger study will aim to further explore these findings.

There are limitations to this work. Polymorphism at codon 129 of the prion protein gene is the major known modifier of disease progression in sCJD and many other prion disease subtypes.^36 37^ As with most clinical studies of prion disease, codon 129 methionine homozygotes are under-represented in this study—a generally precipitous disease course means they are often assessed at very advanced stages of neurodisability. However, the absence of differential performance of the scales in different codon 129 groups suggests that a lower proportion of methionine homozygotes has not biased their development.

Both Motor and Cognitive Scales demonstrated expected floor effects, at mean MRC scores of 3.5 and 3.9/20, respectively. Patients at this advanced level of disability are generally bedbound, with limited verbal or physical communication,^7^ and prognosis at this disease stage very limited.^38^ However, there is considerable variation in the rate at which this stage is reached in patients with sCJD.^39^ Given the intended scale use in routine neurological practice and future clinical trials, we believe that attempting to stratify motor or cognitive function at this advanced disease stage is unlikely to be clinically useful.

We used Rasch methodology to develop two novel clinical rating scales assessing progressive motor and cognitive dysfunction in prion disease, and a composite Examination Scale. These address many of the obstacles involved in assessing clinical change in rapidly progressive, multisystem dementias, and may prove useful in future clinical trials, prognostic modelling and feedback as part of routine clinical care. A similar approach could be employed for other rapidly progressive dementias or comparable multisystem neurological conditions.

## Data Availability

Data are available on reasonable request. Anonymised data are available on reasonable request to the corresponding author.
