# Supplementary figures and images for: Development of novel clinical examination scales for the measurement of disease severity in Creutzfeldt-Jakob disease
Source: J Neurol Neurosurg Psychiatry. 2022 Jan 12;93(4):404–12. doi: 10.1136/jnnp-2021-327722 (PMC8921594; doi:10.1136/jnnp-2021-327722)

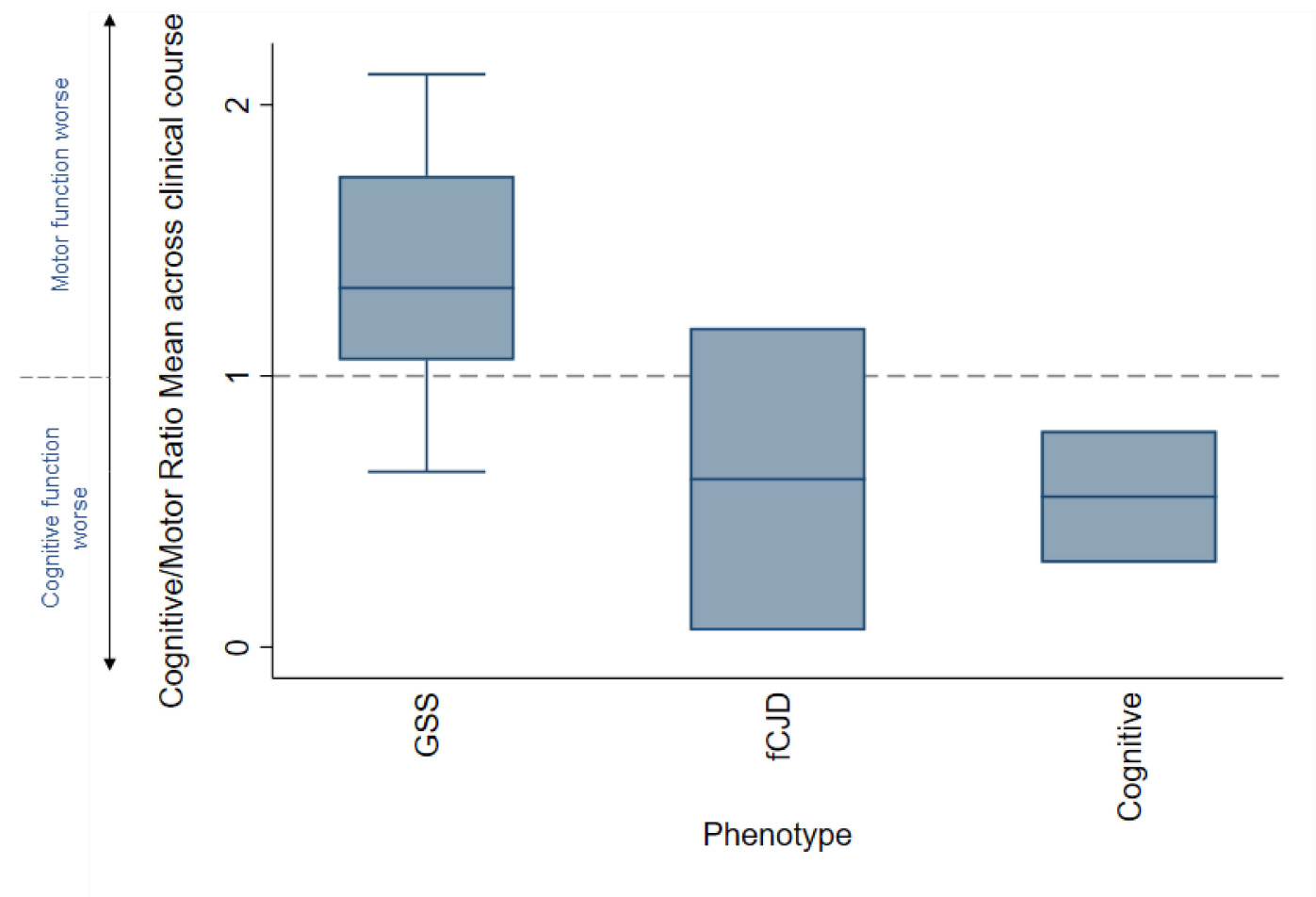

Supplement: Supplementary data [file jnnp-2021-327722supp001.pdf]
